# Case Report: Recurrent intraocular pressure elevation during hemodialysis in a patient with pseudoexfoliation glaucoma

**DOI:** 10.3389/fopht.2025.1658649

**Published:** 2025-09-29

**Authors:** Joshua Eli Herman, Pushpinder Kanda, Ayub Akbari, Deeksha Kundapur, Soumya Podury, Januvi Jegatheswaran

**Affiliations:** 1 Department of Ophthalmology, University of Ottawa, Ottawa, ON, Canada; 2 Division of Nephrology, Department of Medicine, University of Ottawa, Ottawa, ON, Canada; 3 Ottawa Hospital Research Institute, Ottawa, ON, Canada; 4 University of Ottawa, Kidney Research Centre, Ottawa, ON, Canada

**Keywords:** glaucoma, hemodialysis, intraocular pressure, ocular dialysis disequilibrium, pseudoexfoliation

## Abstract

**Introduction:**

Intraocular pressure (IOP) elevation during dialysis has been observed in patients with glaucoma. This is thought to result from rapid shifts in plasma osmolality, leading to fluid movement into the anterior chamber, a phenomenon referred to as ocular dialysis disequilibrium. This case highlights a patient with advanced pseudoexfoliation glaucoma who developed recurrent, symptomatic IOP spikes during dialysis, posing management challenges.

**Methods:**

Case report.

**Results:**

A 65-year-old male with advanced pseudoexfoliation glaucoma experienced recurrent left eye pain and vision loss during hemodialysis, with IOP spikes up to mid 50s (mmHg), requiring early dialysis termination. Medical management including topical drops, oral acetazolamide, and dialysis modifications failed to adequately control IOP. The patient later underwent Ahmed glaucoma valve implantation which stabilized IOP (8–13 mmHg), eliminated dialysis-related pain, and allowed return to standard dialysis sessions. At 6 months, visual acuity was 20/80 + 2 OS with IOP well controlled on topical therapy.

**Conclusion:**

This case demonstrates that ocular dialysis disequilibrium can cause symptomatic IOP spikes in glaucoma patients and may be unresponsive to medical therapy alone. Surgical intervention may be necessary for long-term IOP control. Early recognition and interdisciplinary coordination between ophthalmology and nephrology is critical to prevent irreversible vision loss.

## Introduction

Intraocular pressure (IOP) has been reported to increase during hemodialysis (HD), a process used to remove excess fluid, eliminate waste products, and correct electrolyte imbalances in patients with renal insufficiency (1, 2). HD induces rapid shifts in fluid volume, osmolality, and colloid osmotic pressure, all of which can disrupt normal physiologic fluid dynamics (3). These alterations are thought to promote fluid influx into the anterior chamber, potentially elevating IOP. Although not all patients undergoing HD experience IOP spikes, those with glaucoma and compromised aqueous outflow are particularly susceptible to such changes, placing them at greater risk for progressive optic nerve damage (1, 2).

In this report, we describe a pseudophakic patient with advanced pseudoexfoliation glaucoma (PXG) who presented with recurrent episodes of symptomatic elevated IOP during dialysis. The case highlights the complexities of IOP management in this context and explores both medical and surgical strategies for glaucoma control.

## Case

A 65-year-old African male presented two days to our emergency clinic after having significant left eye (OS) pain during hemodialysis. His past medical history included hypertension, insulin-dependent type 2 diabetes with end-stage kidney disease (ESKD) on chronic HD and obstructive sleep apnea. His past ocular history included PXG, proliferative diabetic retinopathy with pan-retinal photocoagulation bilaterally (OU) and pars-plana vitrectomy OS. His ocular medications included travaprost-timolol daily and brinzolamide-brimonidine three times a day (TID) OS.

**Figure 1:**
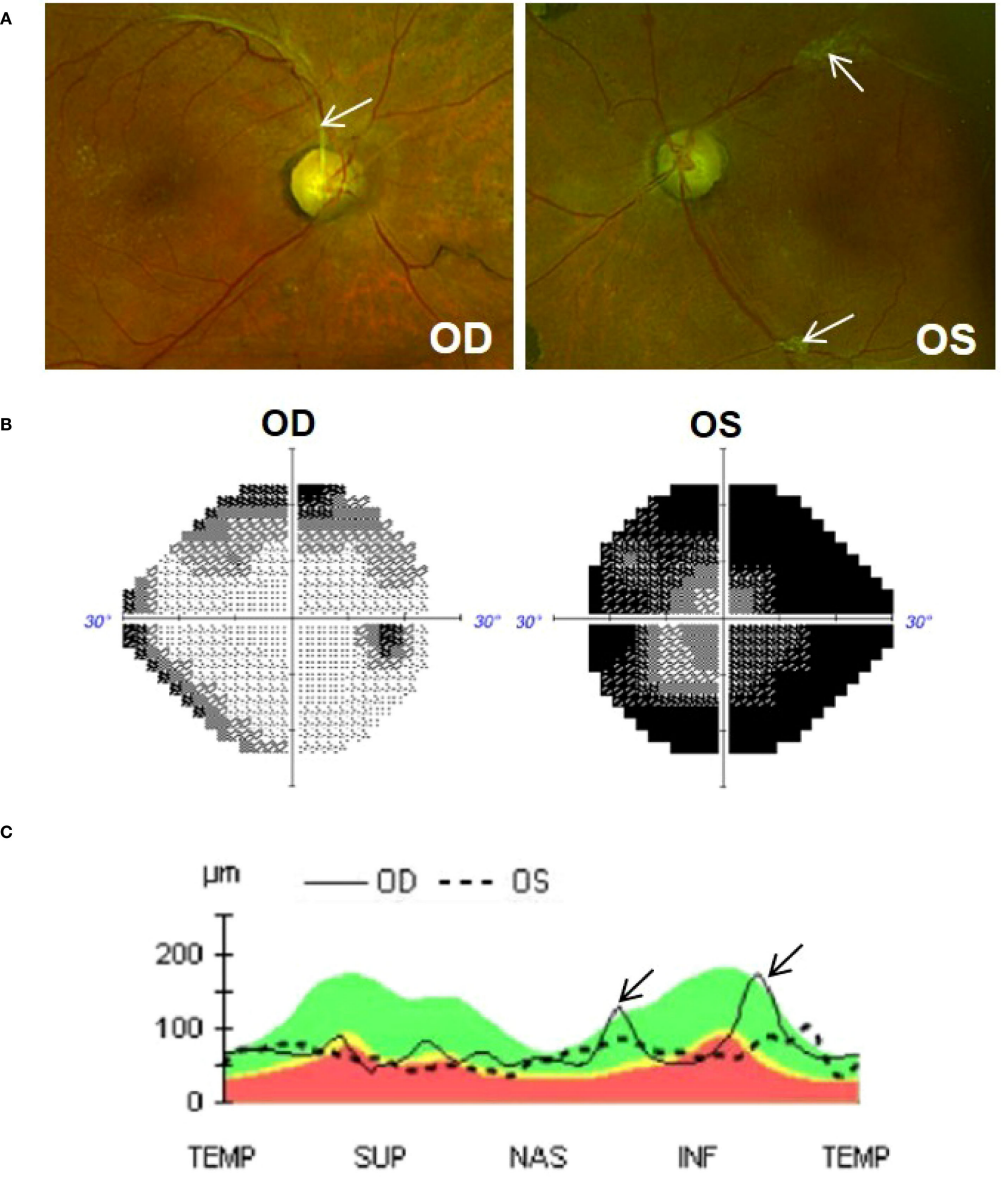
**(A)** Colour fundus photos (Optos) of the right (OD) and left (OS) eye. Optic nerves are cupped (0.8 OD and 0.95 OS) and slightly pale in colour. Tractional fibrous membranes secondary to regressed proliferative diabetic retinopathy can be seen adjacent to vessels (arrows). **(B)** Visual field (Humphery 24-2), showing superior and inferior defect in the OD eye and significant constricted visual field in the OS eye. **(C)** OCT analysis (Cirrus 6000) showing global thinning of the retinal nerve fiber layer (RNFL) in both eyes. Thickened peaks observed in the right eye (arrow) are due to traction exerted by a fibrous membrane. The green shaded area represents normal RNFL (i.e., 90% of normative database falls within this range); yellow represents borderline thinning of RNFL (i.e., 1%-5% of normative database fall within this region); red shaded area represents significant RNFL thinning (i.e., less then <1% of normative database falls within this region).

At initial exam, his best corrected visual acuity (BCVA) was 20/25 OD and 20/60 + 2 OS. IOP measured 7mmHg OD and 9mmHg OS with Goldmann applanation. There was no relative afferent pupillary defect. Slit-lamp biomicroscopy showed no evidence of neovascularization of the iris, angle or retina. He was pseudophakic OU and there was no ocular inflammation. The optic nerves appeared slightly pale and cupped with a cup-to-disc ratio of 0.8 OD and 0.95 OS (**Figure 1A**). Overall, there was no acute ocular pathology at the time of assessment and the patient’s pain had already subsided. Testing of retinal nerve fiber layer (RNFL) with optical coherence tomography (average RNFL thickness of 74µm OD and 65µm OS) and visual field analysis (Visual Field Index (VFI) of 84% OD and 19% OS) revealed that he had moderate-to-advance stage glaucoma in the right eye and very advanced glaucoma in the left eye, **Figures 1B, C**; see **Supplementary Figures 1**-**4** for original unedited OCT and visual field reports.

**Figure 2:**
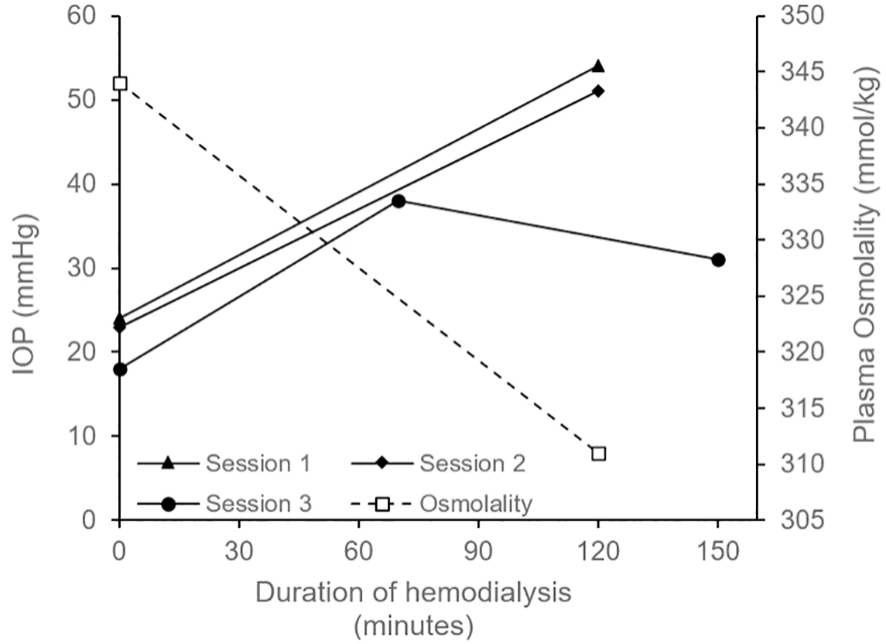
Graph depicting intraocular pressure (IOP) OS measured before the start of hemodialysis (time = 0 minutes) and after its completion (time = 120 minutes for Session 1, 2 and at 130 minutes for Session 3); only one measurement per eye was performed at each time point. In Session 1, patient received acetazolamide 250mg oral 1 hour pre-dialysis. High concentration sodium in the dialysate (140mmol/L) was also started in Session 1 and was continues for all future sessions. In Session 2, patient received 40 mg intravenous mannitol over 2 hours during dialysis. In Session 3, the patient received topical IOP-lowering medications (timolol and dorzolamide-brimonidine) before, during, and after dialysis (see **Supplementary Table 1** for details). Session 3 also included an additional measurement at 70 minutes, and the final IOP reduced to the low 30s mmHg (measured ~20 minutes after completing dialysis). Plasma osmolarity (dotted line) decreased from 344 mmol/kg prior to dialysis to 311 mmol/kg following the session; measurements are from a single collected sample. Goldmann applanation tonometry (GAT) method was used to measure IOP in Session 1 and 2 where iCare rebound tonometry was used for Session 3.

He returned a few weeks later with left eye pain and decreased vision which started 2 hours into dialysis. Immediate evaluation at the time of his symptoms revealed an IOP of 20 mmHg OD and 56mmHg OS by Goldmann applanation, with light perception (LP) vision and significant microcystic corneal edema OS. The anterior chamber was deep with few pigmented cells and there were no keratic precipitates. Gonioscopy showed that the angles were open OU and there was no peripheral anterior synechiae. A therapeutic anterior chamber paracentesis was performed promptly to decrease the IOP. Over the next two dialysis sessions, he again developed left eye pain with IOP spiking from ~20mmHg pre-dialysis to ~54mmHg OS (Goldmann applanation) immediately post-dialysis; **Figure 2** shows IOP trend pre- and post-dialysis at three different sessions (see **Supplementary Table 1** for detailed timeline showing IOP and treatment). Ocular pain during these sessions led to his hemodialysis treatments being terminated two hours early, reducing his usual 4-hour session to 2-hours. The patient described his typical eye pain severity score as 10 during dialysis (0=no pain, 1-3=mild, 4-6=moderate, 7-10=severe).

Given consistent spikes in IOP during dialysis, it was postulated that he had ocular dialysis disequilibrium, a process where elevated IOP is related to rapid shifts in plasma osmolality (see discussion for further details). The nephrology team attempted multiple strategies to minimize a rapid decrease in plasma osmolality in order to reduce the risk of ocular dialysis disequilibrium (see **Supplementary Table 1**). This included using high concentration sodium in the dialysate (140mmol/L as patient’s serum sodium was 128-134mmol/L) and using low efficiency dialysis (blood flow rate 200mL/min and dialysate flow rate of 300mL/min). Another approach to increasing plasma osmolality involved administering 40 mg of intravenous mannitol over the two-hour dialysis session. Pre-treatment with IOP lowering medication was also attempted using oral acetazolamide (125mg-250mg) administered 1 hour before dialysis. Unfortunately, all were ineffective in reducing pain and IOP. Therefore, the ophthalmology team planned for urgent glaucoma surgery which the patient declined.

Given he declined surgery, the nephrology team decided to reduce his dialysis run time from 4 to 2 hours and spread these sessions over 4 weekly visits compared to his previous 3 to ensure that he was not under-dialyzed.

His IOP lowering drops were optimized to lataprostene-bunod nightly, timolol twice a day (BID) and brinzolamide-brimonidine TID. For managing his dialysis related spikes, we implemented a system where he received one drop of timolol and 3 rounds of dorzolamide-brimonidine every 5 minutes at the beginning of dialysis. One hour into dialysis one drop of each medication was applied. At the end of dialysis, again 1 drop of timolol and 3 doses of brimonidine-dorzolamide every 5 minutes was applied. With this regimen, his IOP (iCare rebound tonometry) did not increase into the 50mmHg range (**Figure 2**) and, he was able to better tolerate the 2-hour sessions. Despite these strategies, he would still get weekly attacks of eye pain requiring premature termination, generally close to the 2-hour mark. Of note, the patient had poor compliance to his glaucoma drops and often did not bring his drops to the dialysis unit.

The patient later consented to surgery 2 months after presentation and underwent placement of an Ahmed glaucoma valve in the left eye. His post-operative IOP was 8mmHg OS (Goldmann applanation) at the 1 week follow up while on lataprostene-bunod nightly, timolol BID and brinzolamide-brimonidine TID. Since the surgery, his baseline IOP has ranged from 8-13mmHg OS (Goldmann applanation), see **Supplementary Table 1**. He no longer had any episodes of eye pain during dialysis as such, immediate post-dialysis IOP measurements were not obtained as patient did not have symptoms of pain or vision change. The nephrology team were able to increase his run-time to 4 hours and reduce his visits to 3 times weekly. At 6 months post-operative follow-up, his BCVA was 20/80 + 2 OS with good IOP control on Latanaprostene-bunod once daily and timolol once daily; follow-up visual field (VFI of 83% OD and 17% OS) and OCT (average RNFL thickness of 82µm OD and 75µm OS) were stable (**Supplementary Table 1**, **Supplementary Figures 5**-**7**).

## Discussion

This case demonstrates a clear temporal relationship between HD and symptomatic IOP elevation in a pseudophakic patient with advanced PXG.

Modern dialysis techniques (bicarbonate dialysate, high-flux HD, hemofiltration) have been associated with neutral to lowering IOP effects in literature among the general population (non-glaucomatous eyes), however a recent meta-analysis by Chen et al. showed that narrow angle or impaired outflow glaucoma was associated with intra-dialysis IOP rise (1). Others have reported dialysis related IOP spike in cases of chronic uveitis (4), neovascular glaucoma (5, 6), elevated episcleral venous pressure from abnormal venous blood flow (7) and history of complicated cataract surgery (8). Only two cases have reported similar association with PXG (9, 10). Chen et al.’s meta-analysis also found that IOP spikes during dialysis predominantly occurred in patients receiving acetate-based dialysate (1). Acetate has been largely replaced with bicarbonate in modern dialysis. Although bicarbonate-based dialysis reduces the risk of dialysis-related IOP elevation compared to acetate, it does not eliminate the risk (11, 12). For example, Tawara et al. demonstrated that during bicarbonate-based dialysis, eyes with glaucoma exhibited marked IOP elevations, whereas eyes without glaucoma showed no significant change (2). Our case reflects a similar phenomenon. In PXG, abnormal fibrillar material deposition compromises the trabecular meshwork and Schlemm’s canal leading to reduced aqueous outflow. PXG is often asymmetric (9). In our patient, the left eye had more advanced disease and developed dramatic IOP elevation during dialysis whereas the fellow eye (less severe disease) was relatively spared (IOP only increased by ~3mmHg with the maximum IOP remaining under 20 mmHg). These findings highlight the role of compromised aqueous outflow as a key risk factor for developing intra-dialysis IOP elevation.

### Ocular dialysis disequilibrium

Rapid removal of urea and osmotically active substances such as glucose and sodium during hemodialysis creates an osmotic gradient that promotes fluid movement from extracellular to intracellular space. Clinically, dialysis disequilibrium syndrome is a neurological complication that arises from the rapid removal of urea, leading to fluid shifts from the intravascular space into neurons and resulting in cerebral edema. A similar process, referred to as ocular dialysis disequilibrium, is believed to occur within the eyes (13). It is hypothesized that during dialysis, plasma osmolality drops faster than aqueous humor leading to relatively hyperosmotic intraocular fluid that encourages influx of fluid into the anterior chamber (13). Advanced glaucoma patients with impaired drainage, such as PXG, cannot compensate for this fluid shift leading to increased IOP (1, 9).

In our patient, a single sample of pre-dialysis aqueous humor osmolality was obtained by paracentesis and had an osmolality of 339 mmol/kg; the sample was measured using freezing point depression osmometry within 1 hour of collection). This was similar to his pre-dialysis serum osmolality of 344 mmol/kg. During this session his serum osmolality dropped rapidly from 344 to 311 mmol/kg (**Figure 2**) and was associated with severe eye pain requiring termination of treatment. This supports the ocular dialysis disequilibrium theory where high baseline aqueous humor osmolality combined with a rapid drop in plasma osmolality creates an osmotic gradient that drives fluid into the anterior chamber resulting in elevated IOP.

### Medical management

For medical management, we tried using oral acetazolamide to mitigate dialysis related IOP spikes. However, due to his renal disease, a lower and safer dose of acetazolamide (125mg and 250mg) was used under guidance of the Nephrology team. Unfortunately, addition of oral acetazolamide did not ameliorate the IOP spikes. While a higher dose of 500mg acetazolamide has been shown to lower IOP during dialysis, this was not attempted in our patient due to the potential risk of severe metabolic acidosis (14). The side effect profile of oral acetazolamide (15) makes it unsuitable for long-term IOP management, particularly in patients with end-stage renal disease. Therefore, the focus shifted to strategies aimed at reducing the decline in plasma osmolality during hemodialysis.

Intravenous mannitol administration can increase plasma osmolality and has been shown in a previous case report to minimize the intra-dialysis IOP spike (16). Akin to prior publication (16), we administered 20g of IV mannitol per hour for the first two hours of HD (total 40g of mannitol). Unfortunately, this was not effective as patient had eye pain after two hours of dialysis. A higher dose of IV mannitol was not considered as it is associated with serious side effects including pulmonary edema, exacerbating heart failure, and electrolyte abnormalities (17).

The patient had poorly controlled type 2 diabetes mellitus and his pre-dialysis glucose was often >20 mmol/L. During HD, glucose is removed from blood and causes a decrease in plasma osmolality. There are case reports using IV glucose during HD, in patients with well-controlled or no diabetes, in order to raise plasma osmolality (6, 18). We did not attempt this strategy given the risk of worsening his poorly controlled diabetes. Interestingly, we noticed that the patient tolerated one of his dialysis sessions for 3 hours without symptoms. During this session, the patient was NPO in preparation for glaucoma surgery and his pre-dialysis glucose was 13mmol/L (with full dose of prescribed insulin). We hypothesized that a lower pre-dialysis blood glucose concentration would result in less drastic changes in plasma osmolality. This, in turn, would reduce the osmotic gradient between the aqueous humor and blood, thereby minimizing the increase in IOP during dialysis. As such, we increased the patient’s insulin dose by 10% and added an oral agent (linagliptin) to help manage his diabetes. Despite best efforts, his diabetes remained uncontrolled and his pre-dialysis blood glucose remained elevated (often >20mmol/L). We believe that having better control of blood glucose pre-dialysis may help minimize large IOP spikes during HD in diabetic patients.

### Dialysis modification

We reduced dialysis efficiency by lowering the blood and dialysate flow rates. This approach slows the clearance of solutes from the blood, thereby reducing the rate at which plasma tonicity declines. Slowing the decline in plasma tonicity causes less abrupt osmotic gradient change between the aqueous humor and blood, thereby allowing for more gradual increase in IOP. This was demonstrated in an animal model in 1964 where blood flow rate reduction by 75% minimized IOP spikes (19). We reduced blood flow rate to 200mL/min and dialysate flow rate to 300mL/min as these were the lowest rates that could be safely administered. Despite these changes, the patient developed eye pain two hours after initiation of dialysis. Given long-term reduction in dialysis efficiency can lead to complications of uremia, this strategy was not continued.

We also attempted to increase the dialysate sodium concentration since this increases the plasma sodium concentration and its tonicity. One case report showed that a high dialysate sodium of 150mmol/L minimized IOP elevation during HD (13). Since our patient had hyponatremia at baseline (serum sodium of 128-134mmol/L), a safe dialysate sodium concentration of 140mmol/L was selected. A higher dialysate concentration such as 150mmol/L was avoided in our case as it places hyponatremic patients at increased risk of neurological complications and fluid overload. Unfortunately, the patient continued to have eye pain two hours after initiation of dialysis. Overall, there is no consensus in literature on the management strategies for ocular dialysis disequilibrium. Despite implementing various strategies, the IOP elevation may still continue thus necessitating glaucoma surgery.

### Surgical management

We showed that Ahmed glaucoma valve (AGV) surgery can effectively manage these cases. Our preference for AGV over trabeculectomy was guided by: 1) a more favorable safety profile, including lower rates of postoperative hypotony (20); 2) Given the high initial starting IOP, a controlled reduction of IOP with the AGV (due to it’s valved nature) would avoid risks of intra- or post-operative choroidal hemorrhages due to sudden decompression of the eye; 3) the higher risk of trabeculectomy failure in diabetic eyes (21); 4) prior retinal surgery and use of topical drops had compromised conjunctival health making bleb-based surgery (trabeculectomy) less suitable thus favoring AGV and; 5) trabeculectomy requires more intensive postoperative care (e.g. laser suture lysis and need for needling) which was not suitable for our patient given poor systemic health and inability to make frequent visits to the eye clinic. After glaucoma surgery, the patient reported improved quality of life due to: 1) resolution of dialysis associated ocular pain, 2) a reduced topical medication burden; 3) needing fewer ophthalmology and nephrology visits; before surgery, he was dialyzed four times per week due to shortened sessions from pain but, after surgery, he resumed three times per week; 4) decreased burden for arranging travel requirements to the hospital.

### Functional impact and dialysis-related decline

Coordination between specialties can be challenging and may lead to delays in ophthalmologic assessment. This case emphasizes the importance of timely IOP measurement, as delayed evaluations may fail to detect transient elevations. This is particularly important in patients who remain asymptomatic during dialysis sessions. Consequently, individuals with glaucoma may demonstrate disease progression despite normal IOP readings in clinic, underscoring the need for closer and more frequent monitoring. In our patient, advanced glaucoma with functional loss was already present at the time of referral. Visual field testing showed severe vision loss with a VFI of 19% (Mean Deviation (MD) of -27.24 dB) and moderate-to-severe diffuse RNFL loss (average RNFL of 65µm [Normal is typically ≥80µm]) in the left eye. The right eye had moderate visual field loss (VFI 84% and MD -8.78 dB) and moderate RNFL thinning (average RNFL of 74µm). His disease had likely progressed silently over time due to repeated intradialytic IOP elevations while asymptomatic. As disease progressed (worsening outflow facility) over time, he became symptomatic during dialysis experiencing painful and dramatic IOP spikes. These symptoms ultimately led to ophthalmology referral and glaucoma surgery effectively controlled intradialytic IOP elevation and stabilized his vision. Six months after surgery, progression analysis showed stable visual field (OS: VFI 17%, MD -27.05 dB and OD: VFI 83% and MD -8.99 dB) and stable RNFL (average RNFL OS: 75µm and OD: 82µm). Without timely intervention, further progression and irreversible visual impairment would have been likely.

### Limitations

Limitations of this report include its single-case design, variability in instruments used and operators used to measure IOP, single aqueous and blood sample used for osmolality measurement, limited time points (at least three sessions) showing pre- and intra-dialysis IOP trend, the patient’s poorly controlled diabetes, poor drop compliance, and the presence of multiple concurrent interventions. Also, corneal edema likely resulted in the underestimation of IOP during dialysis-related IOP spike however, this limitation does not change the clinical interpretation i.e., the patient experienced markedly elevated IOP that necessitated active management. Lastly, while the case supports the disequilibrium hypothesis mentioned above, definitive causal physiology remains unproven from a single case. Overall, despite these limitations, the case clearly demonstrates that intra-dialysis IOP spikes can occur in the setting of aqueous outflow obstruction caused by PXG, and that these spikes can be effectively resolved following glaucoma surgery.

## Conclusion

In summary, we present a case of recurrent IOP elevation during dialysis in a patient with advanced pseudoexfoliation glaucoma resulting in pain, vision loss, and early termination of dialysis sessions. Management required a combination of topical and systemic IOP-lowering therapies along with dialysis modifications. The case highlights the importance of timely surgical intervention when medical management is inadequate.

## Data Availability

The original contributions presented in the study are included in the article/**Supplementary Material**. Further inquiries can be directed to the corresponding author.
