# Supplementary material for: Case Report: Recurrent intraocular pressure elevation during hemodialysis in a patient with pseudoexfoliation glaucoma
Source: Front Ophthalmol (Lausanne). 2025 Sep 29;5:1658649. doi: 10.3389/fopht.2025.1658649 (PMC12515614; doi:10.3389/fopht.2025.1658649)
Supplement: Supplementary file 1 [file DataSheet1.pdf]

Supplementary Figures:

Case Report: Recurrent Intraocular Pressure Elevation During Hemodialysis In A Patient With Pseudoexfoliation Glaucoma

Joshua Eli Herman, MBA., MD[1†], Pushpinder Kanda, MD., PhD.[1†], Ayub Akbari, MSc., MD.,[2,3,4] Deeksha Kundapur MD[1], Soumya Podury MD[1], Januvi Jegatheswaran, MD[2,3]

1. Department of Ophthalmology, University of Ottawa, Ottawa, ON, Canada
2. Division of Nephrology, Department of Medicine, University of Ottawa, Ottawa, ON, Canada
3. Ottawa Hospital Research Institute, ON, Canada
4. Kidney Research Centre, ON, Canada

[†]These authors contributed equally to this work and share first authorship

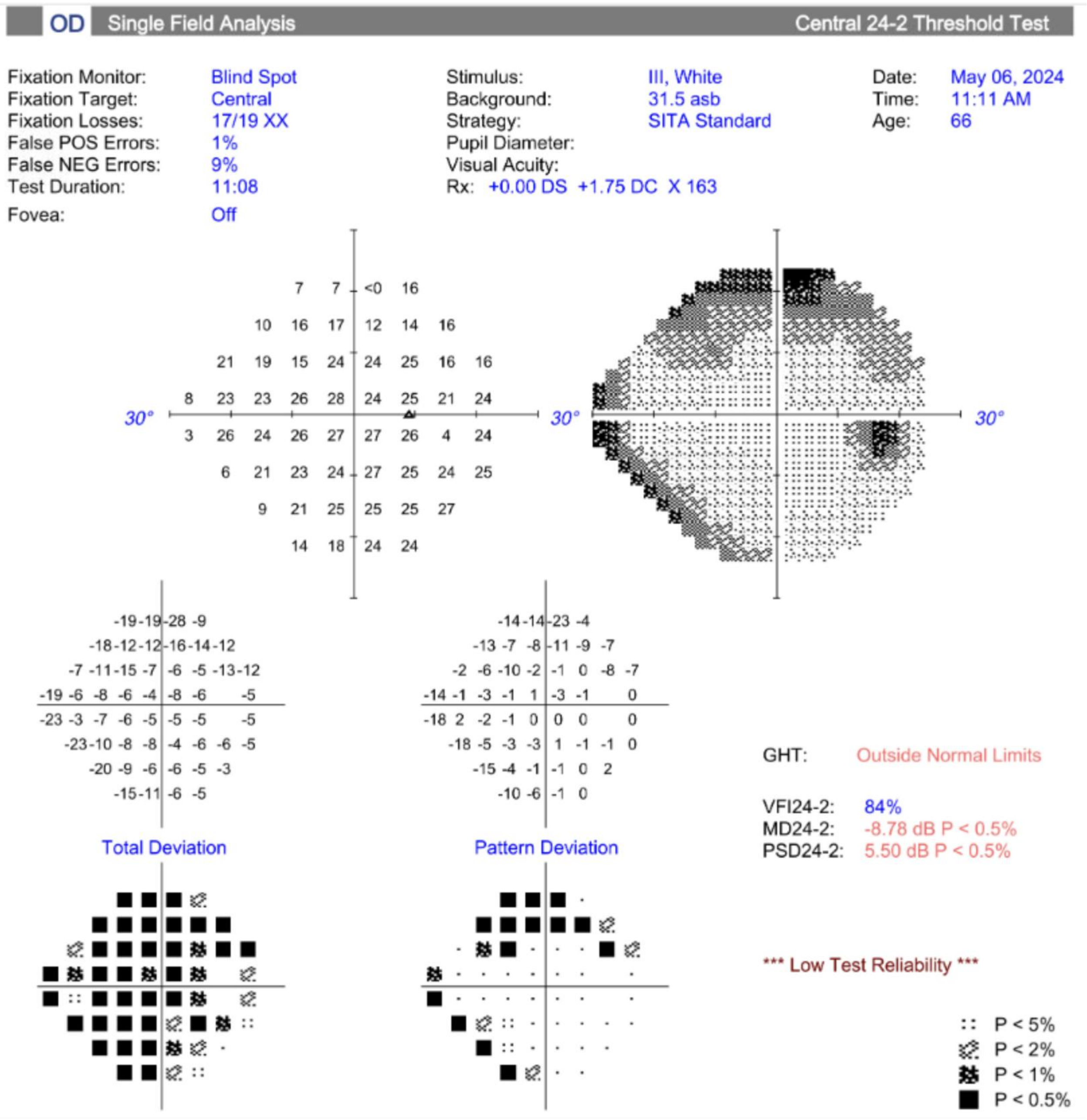

Supplementary Figure 1: Humphery visual field of the OD eye (May 6, 2024).

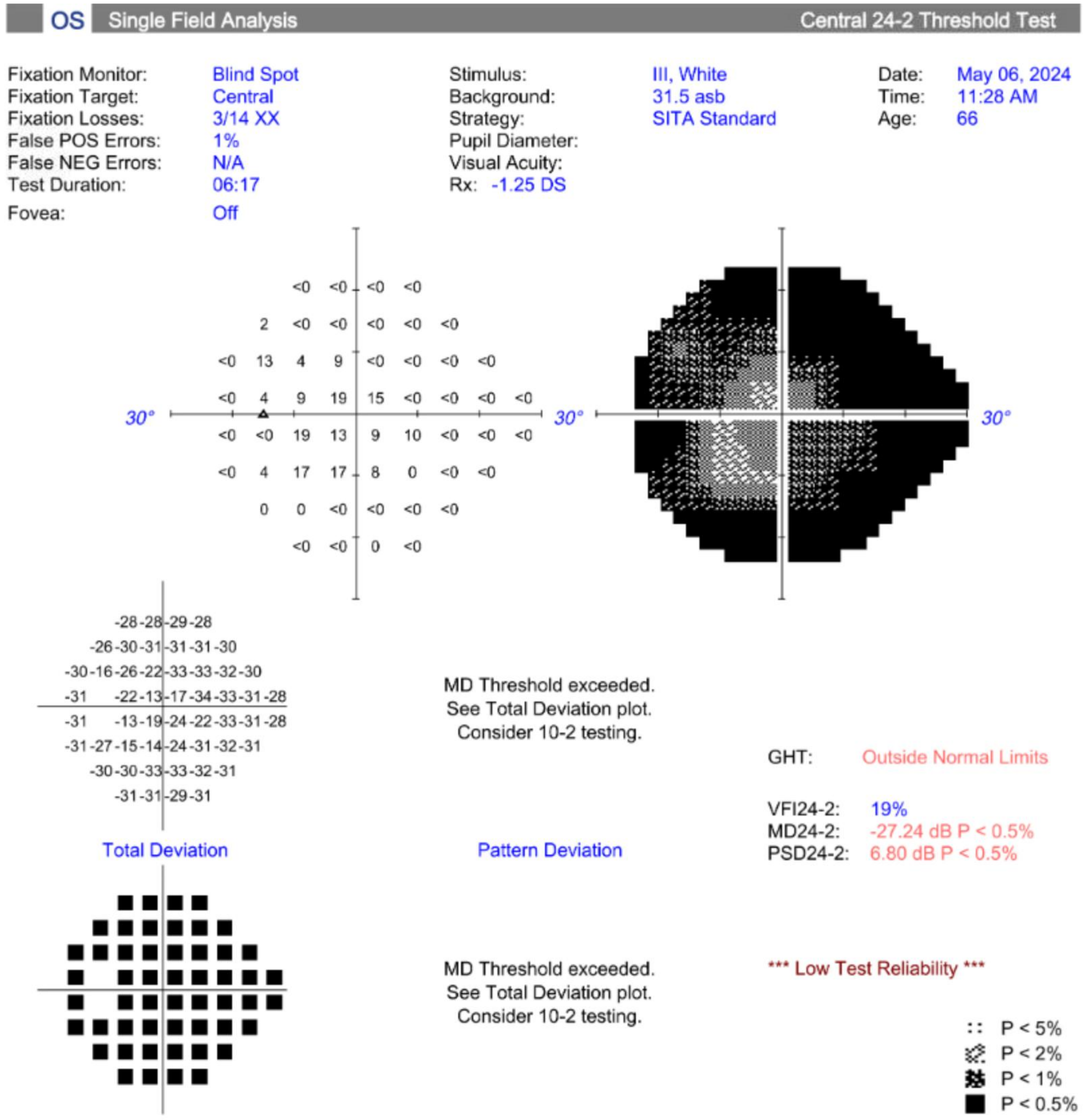

Supplementary Figure 2: Humphery visual field of the OS eye (May 6, 2024).

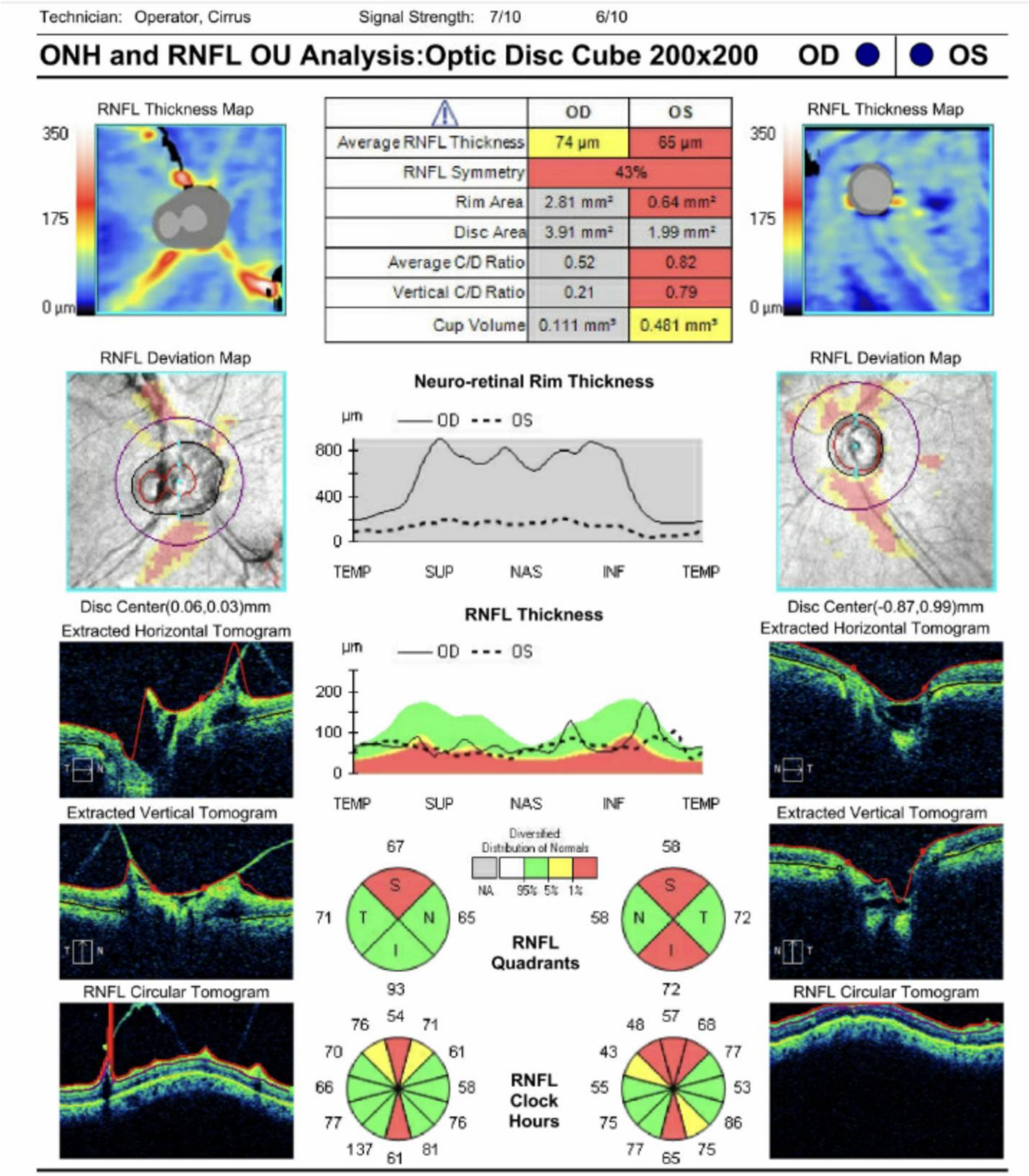

Supplementary Figure 3: Cirrus OCT RNFL (June 4, 2024).

OD:

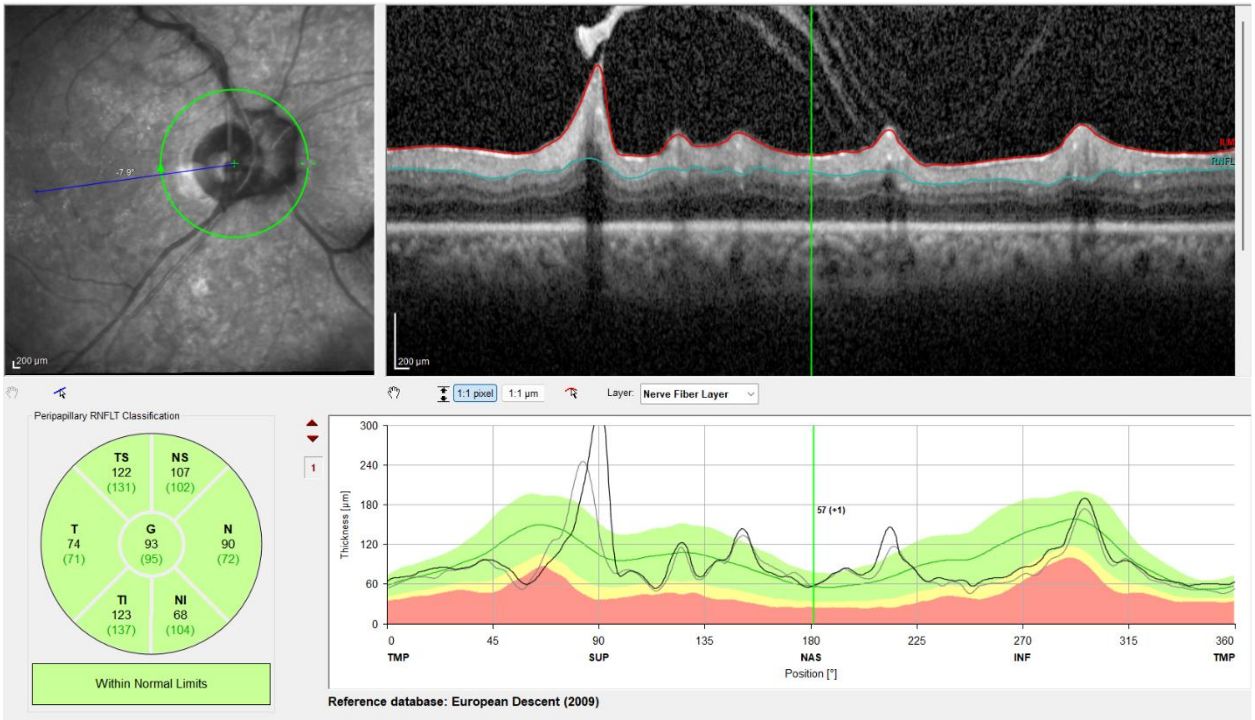

OS:

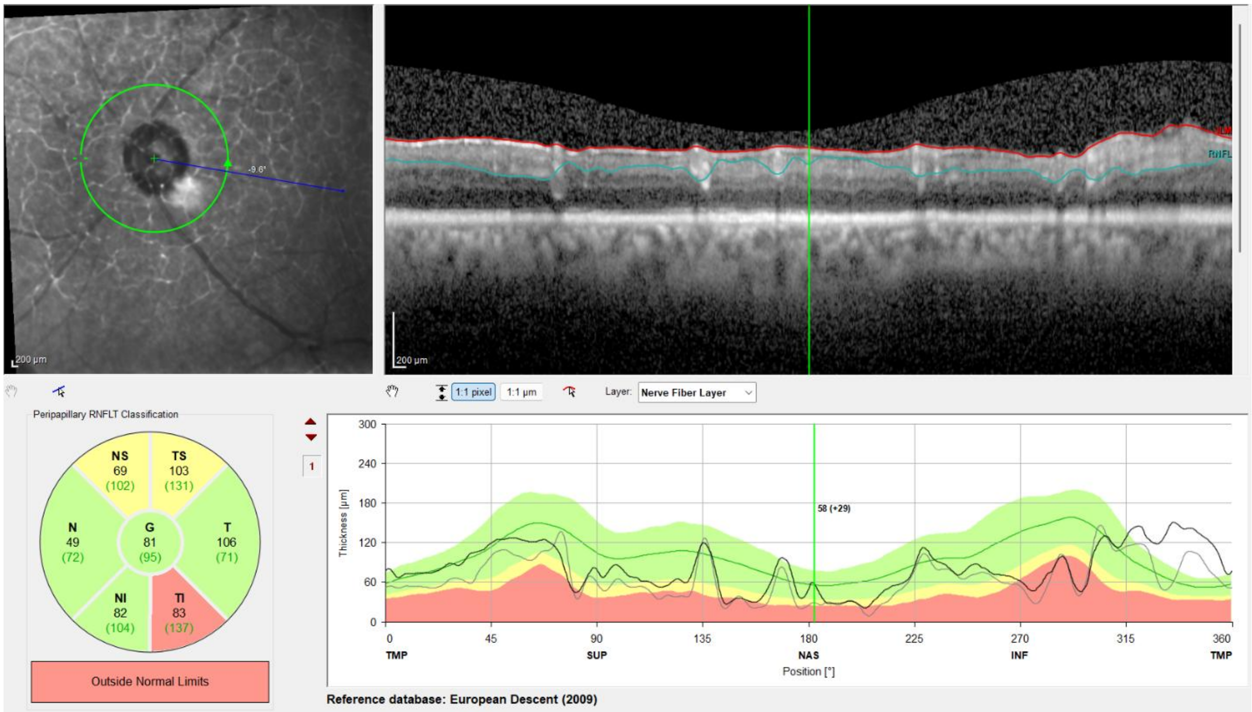

Supplementary Figure 4: Heidelberg Spectralis OCT RNFL (May 6th, 2024). Top=OD eye, Bottom= OS eye.

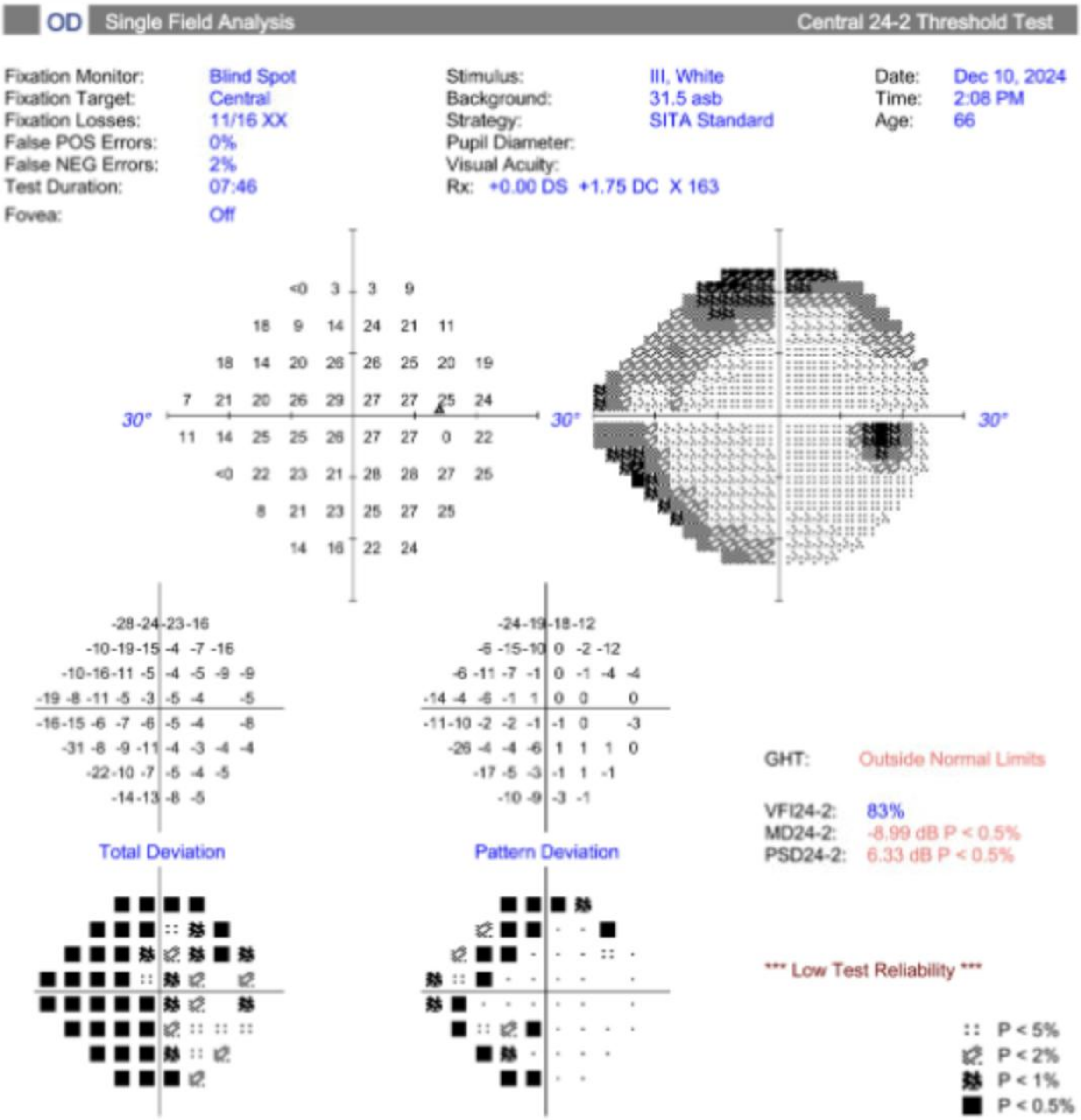

Supplementary Figure 5: Humphery visual field of the OD eye (December 10, 2024). Stable visual field compared to May 6, 2024.

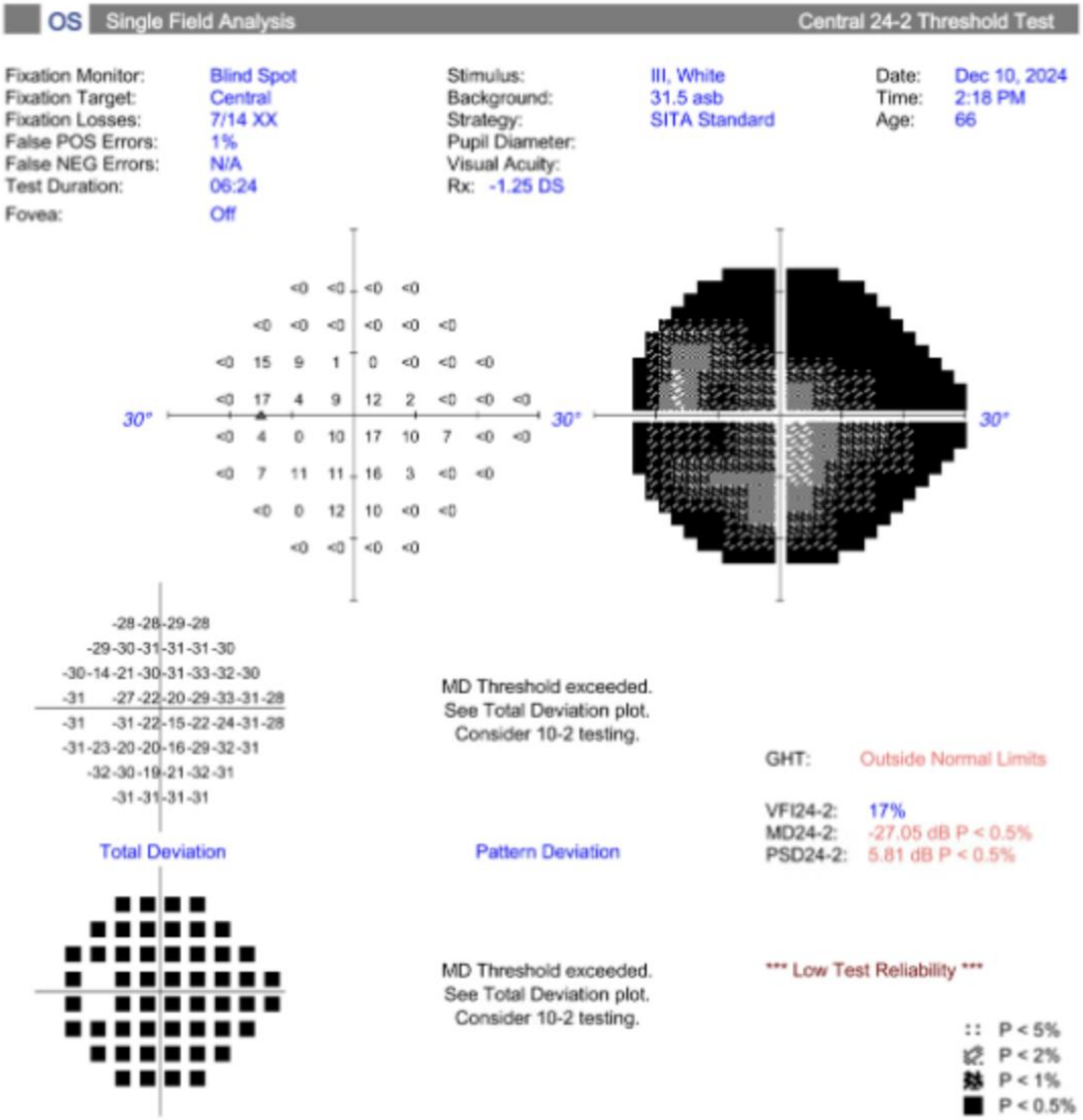

Supplementary Figure 6: Humphery visual field of the OS eye ~6 months post glaucoma surgery of the OS eye (December 10, 2024). Stable visual field compared to May 6, 2024.

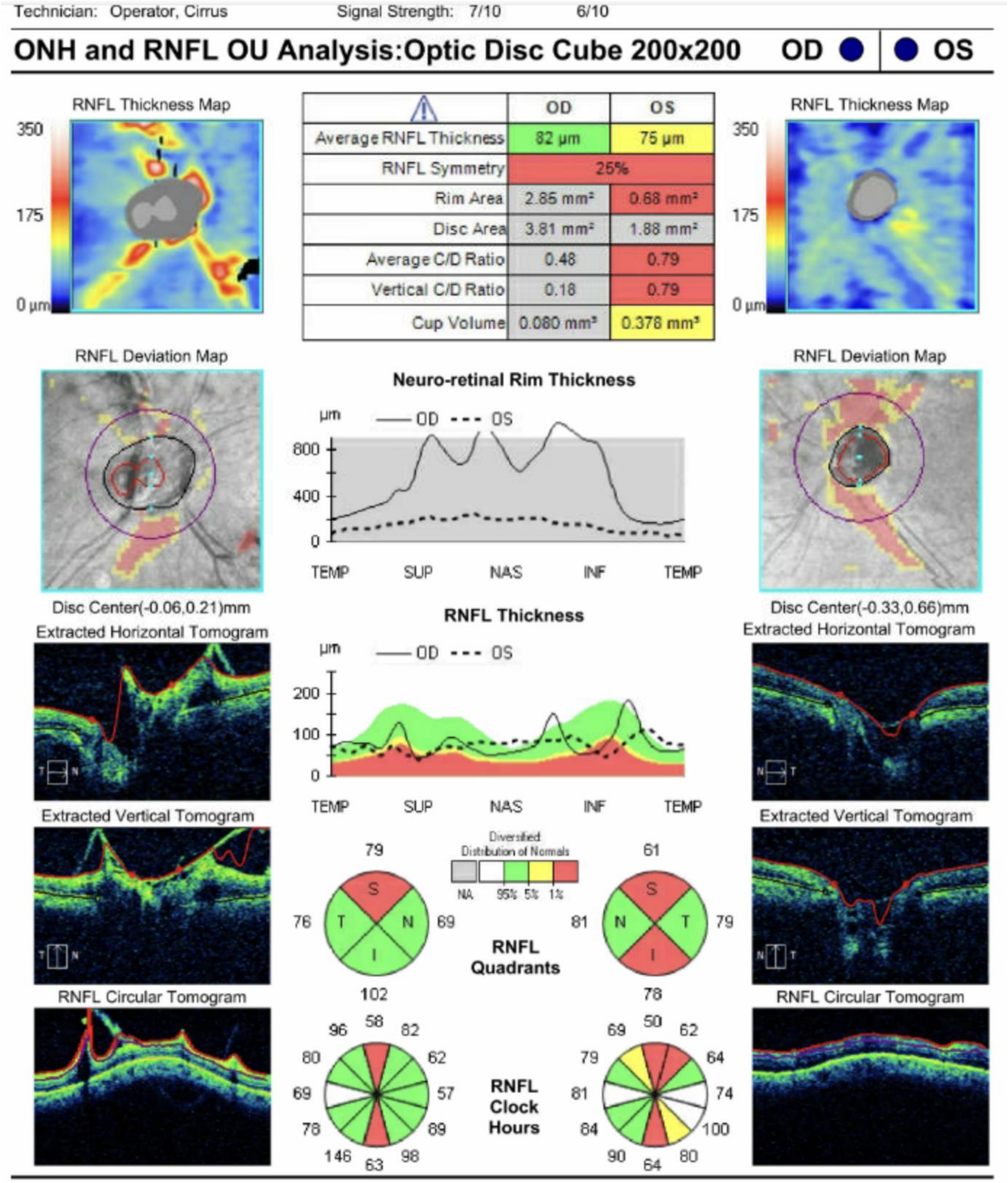

Supplementary Figure 7: Cirrus OCT RNFL (September 6, 2024). Stable OCT compared to June 4, 2024.
