# Supplementary material for: Case Report: Recurrent intraocular pressure elevation during hemodialysis in a patient with pseudoexfoliation glaucoma
Source: Front Ophthalmol (Lausanne). 2025 Sep 29;5:1658649. doi: 10.3389/fopht.2025.1658649 (PMC12515614; doi:10.3389/fopht.2025.1658649)
Supplement: Supplementary file 2 [file DataSheet2.pdf]

Supplementary Table 1: Timeline showing IOP, treatment and dialysis modifications.

Date	Pre-Dialysis IOP (mmHg)	Post-Dialysis IOP (mmHg)	Ocular sign and symptoms	Additional treatment, investigations and dialysis modifications
April 20	N/A	20 OD, 56 OS (GAT after ~2 hours of HD)	OS ocular pain and blurry vision started ~2 hours into dialysis and session stopped early<br><br>OS cornea edema noted on exam	Travaprost-Timolol once daily and Brinzolamide-brimonidine TID OS (topical therapy remained unchanged from this date onward unless specified otherwise).<br><br>Anterior chamber paracentesis after dialysis
May 2	N/A	16 OD, 44 OS (GAT after 2 hours of HD)	OS ocular pain started ~2 hours into dialysis and session stopped early<br><br>OS cornea edema noted on exam	Anterior chamber paracentesis after dialysis
May 4	N/A	N/A	OS ocular pain started ~3 hours into dialysis and session stopped early	Acetazolamide 125 mg oral 1 hour pre-dialysis
May 6	N/A	N/A		HVF 24-2, OCT (Heidelberg Spectralis) and fundus photos (Topcon) (see Figure 1 and Supplementary Figures)<br><br><u>HVF parameters:</u><br>OD: VFI 84%, MD -8.78dB, PSD 5.50 dB<br>OS: VFI 19%, MD -27.24 dB, PSD 6.80 dB<br><br><u>OCT RNFL thickness parameters:</u><br>OD: global average RNFL thickness= 93μm, supratemporal RNFL=122μm, inferotemporal RNFL= 123μm<br><br>OS: global average RNFL thickness= 81μm, supratemporal RNFL=103μm, inferotemporal RNFL= 83μm
May 7	N/A OD, 24 OS (GAT)	20 OD, 54 OS (GAT after 2 hours of HD)<br><br>**See Session 1 in Figure 2**	OS ocular pain ~2 hours into dialysis and session stopped early	**Acetazolamide** 250mg oral 1 hour pre-dialysis<br><br>**Started high concentration sodium** in the dialysate (140mmol/L) and was continued for all HD sessions after this point<br><br>Acetazolamide 250mg oral after completing dialysis

				Anterior chamber paracentesis after dialysis
May 9	10 OD, 23 OS<br>(GAT)	13 OD, 51 OS (GAT after 2 hours of HD)<br><br>**See Session 2 in Figure 2**	OS ocular pain ~2 hours into dialysis and session stopped early<br><br>OS cornea edema noted on exam	**40 mg IV mannitol** over 2 hours during dialysis<br><br>Anterior chamber paracentesis after dialysis
May 11	N/A OD, 19 OS	N/A	No ocular pain during dialysis (completed full 4 hours of dialysis)	Anterior chamber paracentesis just prior to starting dialysis (not a viable long-term treatment thus only performed once)<br><br>**Started low efficiency dialysis** (blood flow rate 200mL/min and dialysate flow rate of 300mL/min)
May 13	13 OD, 26 OS<br>(GAT)	Did not come the eye clinic	OS ocular pain within ~ 3 hours of dialysis and session stopped early	**Anterior chamber paracentesis** done pre-dialysis for measuring **Osmolality** (339 mmol/kg)<br><br>Continued low efficiency dialysis (blood flow rate 200mL/min and dialysate flow rate of 300mL/min)
May 15	N/A	N/A	Ocular pain started within ~3.5 hours of dialysis and session stopped early	Continued low efficiency dialysis (blood flow rate 200mL/min and dialysate flow rate of 300mL/min). Stopped doing low efficiency after this day due to concerns for underdialysis.<br><br>Blood sample collected for **Osmolality measurement** pre-dialysis (344 mmol/kg) and post-dialysis (311 mmol/kg)
May 18	10 OD, 18 OS (iCare rebound tonometer)	16 OD, 38 OS (iCare rebound tonometer after ~70 minutes of HD)<br><br>10 OD, 31 OS (iCare rebound tonometer measured 20 minutes after completing a 2 hours and 10 minutes HD session)<br><br>**See Session 3 in Figure 2**	No ocular pain<br><br>OS very mild cornea edema noted on exam	**<u>Drop strategy during dialysis</u>**:<br><br><u>Just prior to starting dialysis:</u><br>Timolol 1 drop OU, Brimonidine 1 drop every 5min for 3 doses, Dorzolamide 1 drop every 5min for 3 doses<br><br><u>At 1 hour of dialysis:</u><br>1 drop of Timolol, Brimonidine, and Dorzolamide OU<br><br><u>At end of dialysis:</u><br>Brimonidine every 10min for 3 doses, Dorzolamide 1 drop every 10 min for 2 doses

		Because of recurrent ocular pain developing after ~2 hours of dialysis, sessions were shortened to 2 hours in duration, necessitating an increase in frequency to 4 sessions per week instead of the 3		
May 18-July 8		Continued 2 hours of dialysis 4 times per week	No ocular pain (patient declined longer dialysis sessions due to ocular pain)	Cirrus OCT RNFL (June 4, 2024), see Figure 1 and Supplementary Figures.<br><br><u>OCT RNFL thickness parameters:</u><br>OD: average RNFL thickness= 74μm, superior RNFL=67μm, inferior RNFL= 93μm<br><br>OS: average RNFL thickness= 65μm, superior RNFL=58μm, inferior RNFL= 72μm<br><br><u>Eye drops changed to:</u><br>Latanaprostene-bunod once daily OU, timolol BID OU, brinzolamide-brimonidine TID OS<br><br>Drop strategy during dialysis was inconsistently performed as paitent often forgot to bring his drops during the sessions<br><br>No further dialysis modifications were made from this date onwards: continued high concentration sodium in the dialysate (140mmol/L) for 2 hours session 4 times per week<br><br>Increased long-acting insulin from 28 Units to 32 Units (May 18) to help improve blood glucose control<br><br>Added linagliptin (May 22) to help improve blood glucose control
July 8	Patient underwent left eye **glaucoma surgery** (Ahmed glaucoma valve implant)			
July 9	N/A OD, 5 OS (GAT)	N/A	No ocular pain during the 2 hours dialysis session	

July 16	1-week post-surgery IOP: N/A OD, 8 OS (GAT)
July 18	No ocular pain with increased dialysis session to 3 hours
Aug 8	1-month post-surgery IOP: 10 OD, 10 OS (GAT)
Aug 13	**Dialysis session increased to 4 hours and 3 times per week** with no ocular pain
Sept 6	Cirrus OCT RNFL shows no progression of glaucoma (See Supplementary Figures).<br><br>OCT RNFL thickness parameters:<br>OD: average RNFL thickness= 82µm, superior RNFL=79µm, inferior RNFL= 102µm<br>OS: average RNFL thickness= 75µm, superior RNFL=61µm, inferior RNFL= 78µm
Dec 10	6-month post-surgery IOP: 13 OD, 13 OS (GAT)<br><br>No eye pain during dialysis<br><br>Eye drops changes to: Latanaprostene-bunod once daily OU, timolol once daily OU<br><br>HVF 24-2 shows no progression of glaucoma (see Supplementary Figures)<br><br>HVF parameters:<br>OD: VFI 83%, MD -8.99dB, PSD 6.33 dB<br>OS: VFI 17%, MD -27.05 dB, PSD 5.81dB

BID= Two times per day, GAT= Goldmann application tonometry, HD= Hemodialysis, HVF= Humphery visual field, IOP= Intraocular Pressure, IV= Intravenous, MD= Mean deviation, N/A= Not available, OCT= Optical coherence tomography, OD= Right eye, OS=Left eye, OU= Both eyes, PSD= Pattern Standard Deviation, RNFL= Retinal nerve fiber layer, TID= Three times per day, VFI= Visual Field Index
